# Structural and Barrier Properties of Compatibilized PE/PA6 Multinanolayer Films

**DOI:** 10.3390/membranes11020075

**Published:** 2021-01-20

**Authors:** Quentin Lozay, Quentin Beuguel, Nadège Follain, Laurent Lebrun, Alain Guinault, Guillaume Miquelard-Garnier, Sylvie Tencé-Girault, Cyrille Sollogoub, Eric Dargent, Stéphane Marais

**Affiliations:** 1 Normandie University, UNIROUEN Normandie, INSA Rouen, CNRS, PBS, 76000 Rouen, France; Lozay.quentin@yahoo.fr (Q.L.); nadege.follain@univ-rouen.fr (N.F.); laurent.lebrun@univ-rouen.fr (L.L.); 2 Normandie University, UNIROUEN Normandie, INSA Rouen, CNRS, GPM, 76000 Rouen, France; eric.dargent@univ-rouen.fr; 3 Laboratoire PIMM, Arts et Metiers Institute of Technology, CNRS, Cnam, HESAM Universite, 151 Boulevard de l’Hopital, 75013 Paris, France; quentin.beuguel@ensam.eu (Q.B.); alain.guinault@lecnam.net (A.G.); guillaume.miquelardgarnier@lecnam.net (G.M.-G.); Sylvie.girault@ensam.eu (S.T.-G.); cyrille.sollogoub@lecnam.net (C.S.)

**Keywords:** PE/PA6 multinanolayer films, barrier properties, forced assembly coextrusion process, interphase, mechanical properties, serial model of diffusion

## Abstract

The barrier performance and structural lightening of organic materials are increasingly desired and constitute a major challenge for manufacturers, particularly for transport and packaging. A promising technique which tends to emerge in recent years is that of multinanolayer coextrusion. The advantage is that it can produce multilayers made of thousands of very thin layers, leading to new properties due to crystalline morphology changes induced by confinement. This paper is focusing on the study of multinanolayered films with alternated polyethylene (PE), compatibilizer (PEgMA) and polyamide 6 (PA6) layers and made by a forced assembly coextrusion process equipped with layer multiplying elements (LME). PE/PA6 multilayer films consisting of 5 to 2049 layers (respectively 0 to 9 LME) were successfully obtained with well-organized multilayered structure. The evolution of the morphology and the microstructure of these two semi-crystalline polymers, when the thickness of each polymer layer decreases from micro-scale to nano-scale, was correlated to the water and gas transport properties of the PE/PA multilayers. The expected improvement of barrier properties was limited due to the on-edge orientation of crystals in very thin PE and PA6 layers. Despite this change of crystalline morphology, a slight improvement of the gas barrier properties was shown by comparing experimental results with permeabilities predicted on the basis of a serial model developed by considering a PE/PA6 interphase. This interphase observed by TEM images and the on-edge crystal orientation in multilayers were evidenced from mechanical properties showing an increase of the stiffness and the strength.

## 1. Introduction

Polymers have been largely used in packaging applications due to their relative ease of processing, their lightness and transparency. In order to meet all the demanding functional requirements of packaging (good gas permeability, high degree of clarity or transparency, improved chemical, heat and impact resistance), polymers are often combined to achieve synergetic materials properties [1,2]. For example, polyethylene (PE) and polyamide 6 (PA6) are classically associated for their respective mechanical (elasticity and flexibility) and barrier (water and gas) properties [3,4,5]. Coextrusion, used to combine several polymers into a single multilayered film made of alternating layers (classically 3 to 5) is a possible route to achieve packaging films [6].

Since the 70s, Dow Chemical Company has developed the forced assembly coextrusion based on the use of layer multiplying elements (LME) which can generate melt flows made of hundreds to thousands of layers [7,8]. Potentially, each layer can then reach a thickness of a few nanometers [9,10]. The induced confinement and interfaces multiplication may lead to improved optical [11] mechanical [12,13] or barrier properties [14,15].

Studies on multinanolayer materials have shown the formation of oriented crystalline structure for well-chosen semi-crystalline polymers due to confinement effect when the layer thickness decreases below typically 100 nm [16]. When inducing in-plane lamellae parallel to the film surface, a significant improvement of the gas barrier properties has been usually obtained. For example, a decrease of oxygen permeability by almost two orders of magnitude for polyethylene oxide (PEO) has been reported in multinanolayered PEO/polystyrene (PS) films with PEO layer thicknesses around 20 nm. For thicker layers (≳100 nm), confined spherulite morphologies that increase the tortuosity for gas diffusion have been observed for high density polyethylene (HDPE) confined against a cyclic olefin copolymer (COC) [17]. In addition to crystal orientation, other parameters may have a strong influence on the barrier properties, such as the crystal phase [18] or the amorphous phase that can be constrained [14,19,20] and/or oriented [21].

In this work, PE/PA6 multinanolayer films were produced by the forced assembly coextrusion process and with the use of a compatibilizer. The morphological, structural and barrier properties resulting of the nanostratified structure alternating PE and PA6 layers were investigated. If PE usually crystallizes in *α*-phase, PA6 exhibits various stable *α*- or *γ*- forms or metastable *β*-polymorphisms based on H-bonds structuration [22]. In the case of composite films based on PA6, it was shown that the nature of the crystalline phase and its orientation play a key role on the gas barrier properties [23]. For these non-compatible polymers PE and PA6, one hydrophobic the other hydrophilic, the use of a compatibilizer [5] such as polyethylene-*g*-maleic anhydride (PEgMA) is essential, in order to ensure adhesion layers between phases and in consequence to keep the additional properties of both polymers [24]. This is crucial in multinanolayered structures possessing thousands of interfaces.

The aim of this work is to study the structural and transport properties of PE/PA6 multinanolayered films, which is a relevant polymer pair for the industrial production of barrier films. The crystalline morphology will be thoroughly investigated and correlated with barrier properties of the films (nitrogen N_2_, oxygen O_2_, carbon dioxide CO_2_) and water (H_2_O). The influence of interphases on the barrier properties will be discussed in the light of the serial model of diffusion resistance.

The novelty of this work is in the designing of new membranes made of alternating hydrophobic and hydrophilic nanolayers (few nanometers in thickness) of high quality in terms of transparency, mechanical and barrier properties. The interest of such a study was to see the influence of the confinement induced in these multinanolayers and the effect of the multiplied interphases between PE compatibilizer and PA6 on the structural and functional properties.

## 2. Materials and Methods

### 2.1. Materials

Polyethylene (PE), referred as Dowlex^®^ 2645 and polyethylene-graft-maleic anhydride (PEgMA), used as compatibilizer, referred as Amplify^®^ TY1353, were supplied by Dow Chemical (Midland, MI, USA). The MA grafted level of PEgMA is <0.25 wt % according to the supplier. Polyamide 6 (PA6), Ultramid^®^B40 was kindly provided by BASF (Ludwigshafen, Germany). More physical polymer properties are available in Supplementary Information.

### 2.2. Coextrusion Process

**Figure 1:**
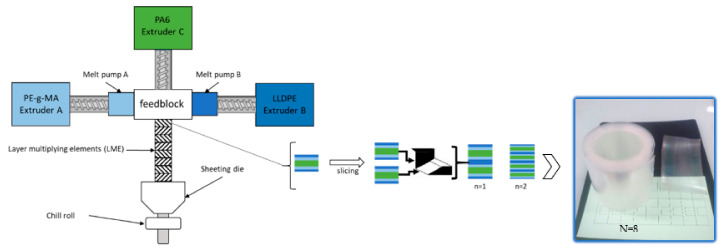
Schematic representation of coextrusion process (**left**); picture of a typical polyethylene (PE)/PEgMA/PA6 multilayer film using 8 LME (**right**).

Before processing, PA6 was dried for 48h under dry air flow at 80 °C. The set-up coextrusion process (Figure 1) used three main extruders. The molten polymers were joined in an A/B/C/B/A coextrusion feedblock at 240 °C in order to obtain a 5-layers polymer flow. Then, the flow passed through layer multiplying elements (LME) placed after the feedblock. Each element divided the flow vertically in two parts and then recombined it horizontally while maintaining the total flow thickness constant. In this configuration, the total number of layers *n_tot_* varies with the number of multiplier elements *N* following Equation (1):

(1) $$n_{tot}=2^{N+2}+1$$

In this work, 0, 5, 8 and 9 LME were used, leading to PE/PEgMA/PA6 multilayers consisting in 5, 129, 1025 and 2049 layers, respectively.

The chill roll temperature was set to 80 °C and the final film thickness was fixed at 100 µm and the PE/PEgMA/PA6 concentration was set to 25/50/25 wt % for all films.

In addition, reference films of each polymer were elaborated with the same final thickness without and with 8 LME. The chill roll temperature was fixed at 50 °C for PE and PEgMA films and at 80 °C for PA6.

### 2.3. Morphological Characterization

Polarized optical microscope (POM) observations were performed using a Zeiss Axio Imager 2 optical microscope equipped with an AxioCam ICC I camera and cross polarizer (Carl Zeiss, Oberkochen, Germany). The 10 µm thick samples were cut at room temperature with a Leica RM225 microtome (Leica, Wetzlar, Germany), using a glass knife with a cutting speed of 1 mm/s. Image acquisition was performed using AxioVision 4.9.1 software and the micrographs were analyzed using Image J software.

Transmission Electron Microscopy (TEM) analysis was performed on FEI Tecnai 12 Biotwin (FEI Compagny, Hillsboro, OR, USA) with a tension of 80 keV and a thermionic electron gun in LaB6 equipped with CCD Erlangshen ES500W camera (Gatan Pleasanton, CA, USA) The different images were treated with Gatan Digital Micrograph software. The samples were sliced at −140 °C, at a thickness of 80 nm with a Leica Ultracut UTC7 ultracryomicrotome (Leica Biosystems, Wetzlar, Germany) equipped with a diamond knife. The cutting speed was set to 1 mm/s.

It was verified, using the method described previously in [25] that the layer thicknesses of the obtained films were consistent with the theoretical thickness with a thickness variation below 15%.

The experimental and calculated data of layers are given in Supplementary Information (Table S2).

### 2.4. Structural Characterization

Differential Scanning Calorimetry analyses were performed in standard aluminum pans during the first heating scan from 25 to 250 °C at 10 °C/min, after indium calibration, using a DSC-Q1000 (TA instrument, New Castle, DE, USA). N_2_ was used as purging gas with a flow of 30 mL·min^−1^. The melting temperature *T_m_* is determined at the maximum of the melting peak and the degree of crystallinity X*_c_* of each polymer is calculated using the theoretical melting enthalpy of 100% crystalline polymer, ~279 J·g^−1^ [26,27] for PE (and PEgMA) and ~240 J·g^−1^ [28] for PA6. In addition, thermo-modulated-DSC (TM-DSC) analyses were done to determine precisely the glass transition temperature of each polymer and particularly for PA6. TM-DSC experiments were performed using Q100 TA Instruments (TA instrument, New Castle, DE, USA) from −20 to 250 °C at 2 °C/min in aluminum pans. The heat-only modulation of ±0.318 °C in a period of 60 s was applied. The glass transition *T_g_* was determined from the reversible signal. Thermal analyses were performed with a mass sample of ~6–8 mg.

In-situ small and wide-angle X-ray scattering (SAXS and WAXS) experiments were performed at different temperatures, in normal direction (ND) on the Swing beamline of the Soleil National French Synchrotron. The monochromator was set to 17 kV (corresponding to a wavelength *λ* of 0.775 Å) and the distance was fixed to 6.22 m and 0.55 m for SAXS and WAXS, respectively. The reciprocal scattering vector values *q* ranging from 0.0055 to 0.24 Å^−1^ for SAXS and from 0.03 to 2.3 Å^−1^ for WAXS. The patterns were recorded at *T* = 25 °C or in temperature range *T* from 25 to 250 °C, at 10 °C/min, using a LINKAM heating stage THMS600. The data were acquired with an exposition time of 1 s every ~0.83 °C. The intensity signal was reduced with a blank diffractogram subtraction. The circular 1D azimuthal integration over all *Ψ* available was done using Foxtrot software.

Simultaneously SAXS and WAXS [29] were also carried out at room temperature on stacked films with a respective thickness of 1, 1.6 and 2.5 mm in normal (ND), transverse (TD) and extrusion direction (ED), in order to optimize the scattered intensity. Since films are thin compared to the beam section (800 μm), stacking of film was necessary for ED and TD. These tests were performed on the Nano-inXider SW (Xenocs, Sassenage, France) system in transmission using CuK α radiation at a wavelength *λ* of 1.54 Å and operating at 50 kV and 0.6 mA. The detector positions were set to 933 mm for SAXS and 78 mm for WAXS, leading to a continuous reciprocal scattering vector values *q* range between 0.01 and 4.2 Å^−1^. The intensity signal was normalized by the stack sample transmission and thickness.

### 2.5. Transport Properties

#### 2.5.1. Gas Permeation

Gas permeation measurements were carried out by the “time-lag” method at 25 °C with a lab-made apparatus [30]. The sample was placed in a permeation cell composed of the upstream and downstream parts. First, before permeation measurement, vacuum was applied during at least 15 h to remove residual gas and water molecules in the sample and the permeation device. After this purge step the upstream compartment was filled with the gas (N_2_, O_2_ or CO_2_) at 4 bar pressure). Due to the driving force created from the pressure gradient on both sides of the film, the gas permeates the film from the upstream to the downstream side. In the downstream side the increase of pressure is recorded as function of time until reaching the stationary state. The permeability coefficient *P* expressed in barrer unit (1 barrer = 10^−10^ cm^3^_(STP)_·cm·cm^−2^·s^−1^·cm·Hg^−1^) was determined from the stationary flux according to:

(2) $$P=\;\frac{J_{st}\;L}{\Delta_{p}}$$

where *L* is the film thickness, $\Delta_{p}$ is the pressure difference between both compartments. The stationary flux $J_{st}$ is obtained from the slope α of the time-lag kinetic curve *pressure = f(t)* at long time:

(3) $$J_{st}=\;\frac{\alpha \;V}{A\;R\;T}$$

With *V* the volume of the downstream cell, *R* the perfect gas constant, *A* the active surface of the sample in contact with gas and T is temperature.

#### 2.5.2. Water Permeation

Water permeation measurements were performed at 25 °C with a lab-build permeameter [31] which consists of a permeation cell separated in two parts by the film to be tested. For the purge step, the permeation cell with the film was swept with dry N_2_ for few hours to achieve a very low water concentration (<2 ppmV). Then, the upstream part was refilled with liquid water (milli Q) while the downstream compartment was continuously swept by ultra-dry nitrogen. Due to the driving force created by the gradient of water concentration gradient, water molecules permeate through the sample thickness from the upstream to the downstream. The permeation flux of water in downstream is then measured as function of time, via a chilled mirror hygrometer (Elcowa^®^, France, General Eastern Instrument), until reaching a constant value that is the steady state. The permeability coefficient (in Barrer) is determined from the stationary flux with Equation (3). All permeation average data were reproducible to ±5%, knowing that reproducibility was checked from at least three measurements for each sample.

### 2.6. Mechanical Properties

The uniaxial tensile properties were determined with an Instron 5543 mono-column traction machine (Instron, Norwood, MA, USA) equipped with ±500 N strength sensor. The samples were bone-shaped in agreement with ISO527 type 3. The samples were stretched at 50 mm/min. The Young’s modulus, tensile strength and elongation at break were deduced from the stress-strain curve and from at least ten samples per films and per directions.

## 3. Results and Discussion

### 3.1. Structural Characterization

#### 3.1.1. Morphology

**Figure 2:**
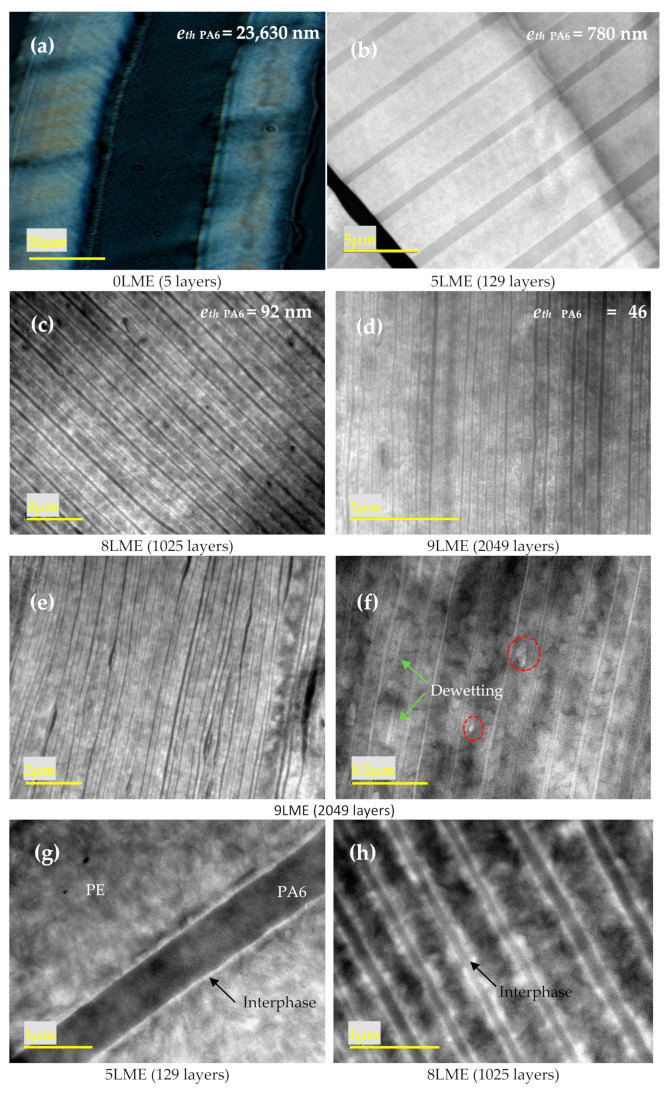
Optical (**a**), TEM (**b**–**f**), images of (**a**–**f**) PE/PEgMA/PA6 multilayer films using 0 (**a**), 5 (**b**,**g**), 8 (**c**,**h**) and 9 (**d**–**f**) multiplying elements.

The typical layered structure of the PE/PEgMA/PA6 assembly obtained for all samples is clearly observed in Figure 2. PE and PEgMA are miscible and at, the processing temperatures, they will interdiffuse during the extrusion residence time, which is confirmed by the fact that only two types of layers can be distinguished: PA6 and PE/PEgMA.

As stated above, the thicknesses of micro- and nanolayers are close to the theoretical values calculated from flow rates (see Supplementary Information, Table S2). We note that there is a slight layer thickness distribution, which may be explained by the viscosity differences between phases (Table S2). Nevertheless, all PE (i.e., merged PE and PEgMA) and PA6 layers are continuous for films up to 9 LME, with layer thicknesses down to 46 nm (Figure 2a–d). Layer breakup appears only for the thinnest layers [10], i.e., *e*th _PA6_ = 46 nm (9 LME film), with an amount of broken layers below 20% (Figure 2e,f).

In Figure 2g,h, the thin white layer clearly observable between PE and PA6 layers was attributed to an interphase. The determination of the interfacial thickness in compatibilized polymer systems (i.e., interphase) is often questionable. From these TEM images, the interphase PE/PA6 appears to be approximatively 30–40 nm thick, which agrees well with the one measured by ellipsometry and TEM (38 nm) by Li et al. [32] on similar PPgMA/PA6 systems. Considering that a copolymer is chemically formed at the PE/PA6 interface during extrusion by the reaction of PEgMA with the PA6 amine end-groups, the size of the interphase can be simply estimated as the end-to-end distance radius (R_0_) of the copolymer in a random coil conformation, which is roughly obtained by adding the average end-to-end distance radius of both polymers (R_0_ ≈ 15 nm for PEgMA and R_0_ ≈ 20 nm for PA6) [33], consistent with the TEM observation.

#### 3.1.2. Crystallization

**Figure 3:**
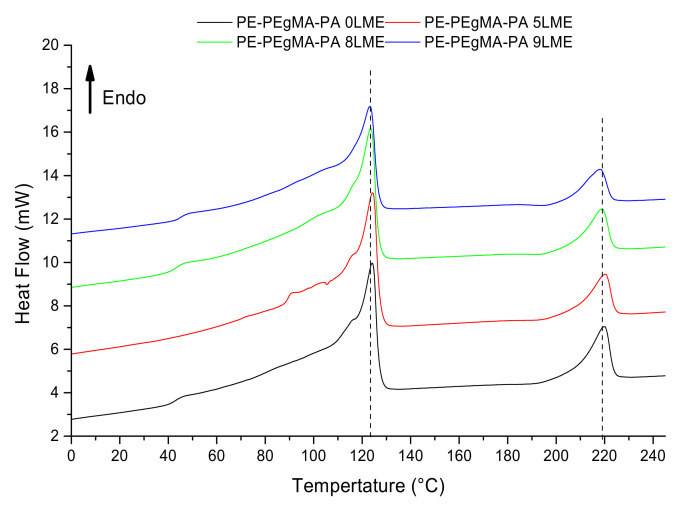
DSC for PE/PEgMA/PA6 multilayer films made by 0 LME (5 layers), 5 LME (129 layers), 8 LME (1025 layers) and 9 LME (2049 layers).

Figure 3 presents the DSC scans for PE/PEgMA/PA6 multilayer films made with different LME number *N* (0, 5, 8 and 9). Similar thermograms are obtained for all the films, on which several thermal events are observable. The first endothermic peak is due to the melting of PEgMA and PE, while the second peak at higher temperature is attributed to the melting of PA6. The melting temperature *T_m_* = 219 °C suggests the presence of γ-phase only [34]. For all systems, the crystallinity ratio X*_c_* is close to 39% for PE-PEgMA (PE) and 28% for PA6 (Table 1). It is worth pointing out that conventional DSC scans are slightly perturbed by secondary melting/crystallization and water evaporation phenomena for PA6, as highlighted by TM-DSC (Supplementary Information, Figure S1).

The crystalline structure of each polymer, their lamellae periodicity and their orientation inside the PE and PA6 layers have been investigated using SAXS and WAXS.

The 0 LME film SAXS and WAXS patterns in ND shows a quasi-isotropic crystal orientation while the ones associated to the 8 LME is highly oriented (Supplementary Information, Figure S2).

The crystalline structures of both semi-crystalline polymers have been reported for about fifty years [35,36,37]. They are summarized in Supporting Information (Table S3) and show many diffraction peaks superimposed for both PE and PA6 phases. In-situ WAXS experiments during a ramp at 10 °C/min enable to observe PA6, switching off PE phase, due to the well separated PE and PA6 melting temperatures.

**Figure 4:**
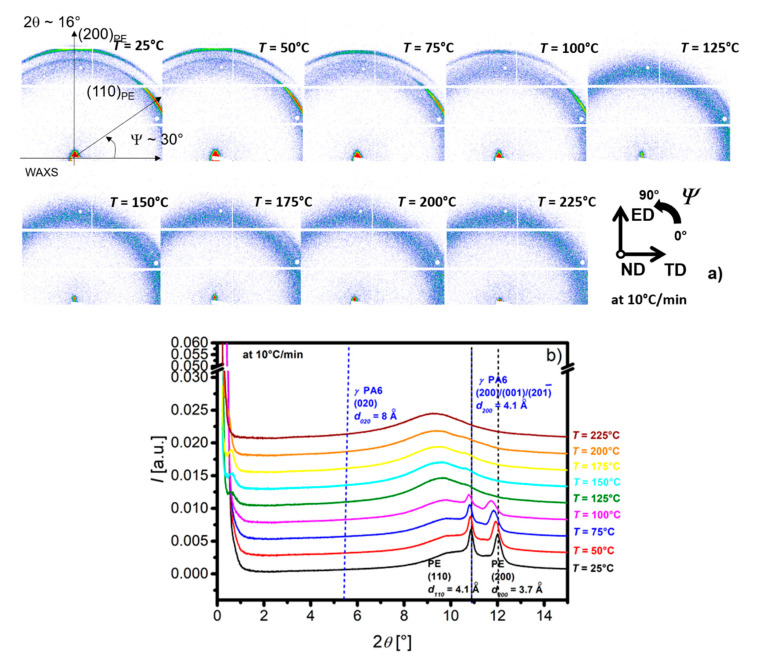
2D WAXS of 8 LME PE/PEgMA/PA6 multilayer films in normal direction (ND) at different temperatures T. (**a**): 2D-patterns, (**b**): 1-D Diffractograms obtained after azimuthal integration.

Figure 4 presents 2D WAXS patterns for the 8 LME at different temperatures ranging from 25 to 225 °C (Figure 4a) and azimuthal integrations (Figure 4b).

At wide angles and room temperature, the diffraction (Figure 4a) intensity is higher for ψ~30° and ψ~90° for the respective first and the second rings. In the center of the 2-D images (at small angles) an anisotropic scattering (ψ~90°) is observed. Both SAXS and WAXS scattering are anisotropic, but the extension of the detector and the recorded q-range are too limited to characterize properly this anisotropy. Regardless of the orientation, the evolution of 1-D azimuthal integration has been monitored during first heating (Figure 4b). The two Bragg peaks at 2θ~11 and 12° correspond to the interplanar distances of the PE (Table S3). In the small angle range, the scattering intensity corresponds to the crystalline lamellar arrangement but cannot be precisely observed due to the *q* range limitation at around 0.03 Å^−1^ (2θ~0.3°). Above the melting point of PE (T > 125 °C), the intense diffusion at low angles disappears as well as the peaks at wide angles due to PE melting. At small angles, a broad peak at 2θ~0.6° (q~0.1 Å^−1^) which shifts towards small angles as the temperature increases is attributed to the PA6 periodic crystalline lamellae. In the wide-angle range, a very small diffraction peak can be seen at 2θ~11° and a shoulder at 2θ~5.5° attributed to the respective interplanar distances d_001_/d_200_/d_20_${}_{\bar{1}}$ = 4.1Å and d_020_ = 8 Å of the PA6 γ-phase (Table S3). These peaks disappear above 225 °C.

The crystalline structure of the PA6 and their evolution during heating have been confirmed using the same in-situ experiment on PA6 reference film (Supplementary Information, Figure S3).

**Figure 5:**
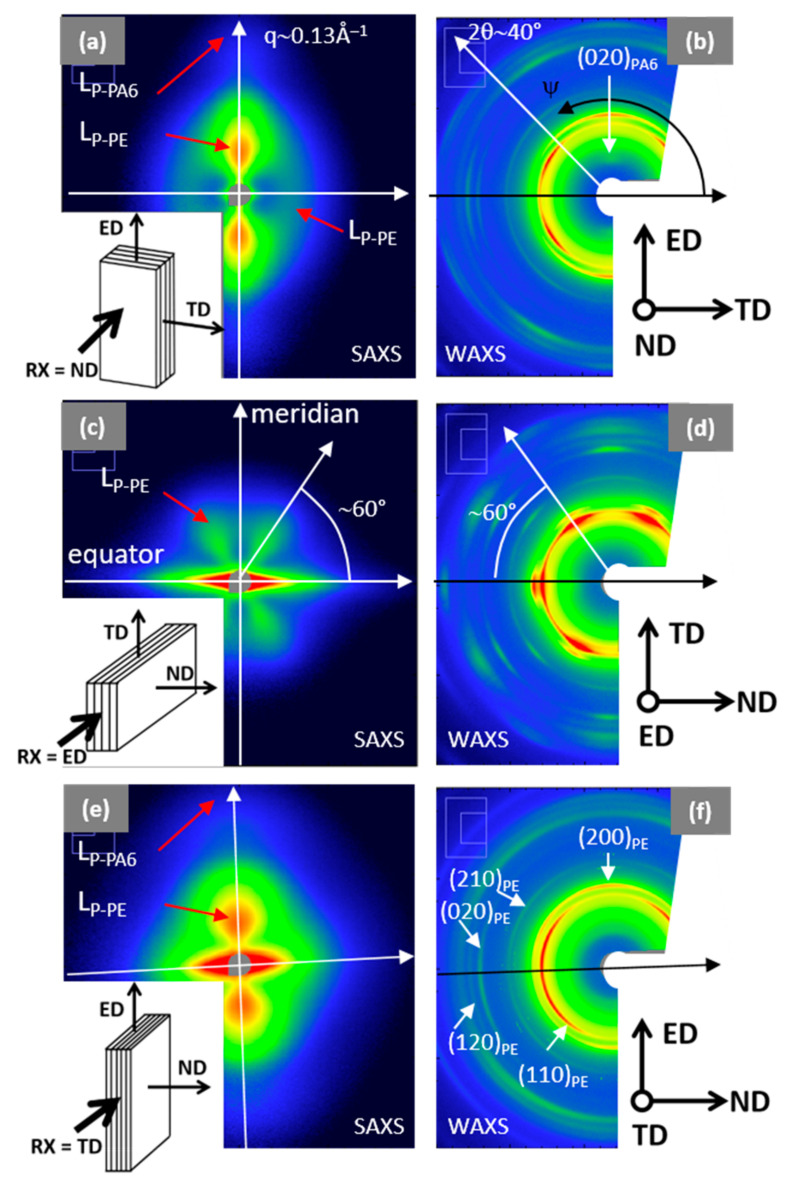
2D SAXS (on left) and WAXS (on right) patterns of 8 LME PE/PEgMA/PA6 multilayer films in (**a**–**b**): normal (ND), in (**c**–**d**): extrusion (ED), (**e**–**f**): transverse (TD) direction. The *q* range in SAXS is 0.01 to 0.13 Å^−1^, the 2θ range in WAXS is 5 to 40°.

In addition to these experiments, simultaneous SAXS-WAXS experiments in the three main directions of the 8 LME extruded multilayer film (ND, ED and TD) have been performed with wide *q* and *ψ* ranges (Figure 5). SAXS images reported on the left side of the Figure 5 correspond to a *q* range from 0.01 Å^−1^ to 0.13 Å^−1^, while WAXS images, on the right side, show a 2θ range between 5 and 40°. The color intensity scales (logarithmic) are adjusted to reflect the true corrected intensity ratio between the different images. 1-D diffractograms at different *ψ* angles are available in Supplementary Information Figure S4 for complementary information.

The very intense linear scattering observed along the equator in ND direction, Figure 5c,e, does not concern the structure or the morphology of the polymers, since it is due to film stacking or interface diffusions. All the SAXS and WAXS patterns are anisotropic. The orientation on SAXS images related to the periodic stacking of the crystalline lamellae is compared to the orientation of the cells axis (Figure 5a–c) in the crystalline lamellae observed on WAXS images. The chain directions of PE and PA6 are c- and b-axis, respectively.

SAXS and WAXS patterns with X-ray beam along ND (Figure 5a,b) and TD (Figure 5e,f) are comparable, in terms of orientation and intensity. SAXS scattering is mainly oriented along the meridian (*ψ* = 90°), i.e., the ED direction. The more intense scattering corresponds to the PE long period (L_P-PE_~210 Å) while the smaller, at higher q, corresponds to the PA6 (L_P-PA6_~65 Å). The main orientation of the PE and PA6 crystalline lamellae is perpendicular to ED, corresponding to the so-called on-edge orientation (i.e., perpendicular to the film surface and to the layer interfaces). The corresponding WAXS pattern (Figure 5b,f) exhibit diffraction arcs localized close to the equator, associated to (hk0) planes of PE. These agree with the main orientation of the PE crystalline lamellae and the *c*-axis chain orientation of the PE along ED. Moreover, in Figure 5a another small broad peak at q~0.03 Å^−1^ is observed along the equator (*ψ* = 0°), corresponding to «isotropic» PE lamellae with L_P_-_PE_~180 Å. A very small peak associated to a diffraction of the PA6 chain direction ((020)_PA6_) is identified along the meridian (Figure 5b). This diffraction is consistent with the on-edge orientation of the PA6 crystalline lamellae. Surprisingly, in the WAXS pictures (Figure 5b,f), the (200)_PE_ diffraction is observed along the meridian (ED direction) and the (110)_PE_ diffraction is highlighted at *ψ* = 30 and 150°, which shows that *a*-axis of PE-crystal is oriented in ED and the *b*-axis in TD, suggesting that *c*-axis is along the ND direction. However, the very intense diffusion observed in SAXS along ND direction (Figure 5e) prevents us from concluding on this point.

Along the ED direction, the SAXS pattern (Figure 5c) exhibits a 4-leaf clover pattern with an intensity two decades lower compared to Figure 5a,e and with maximum peaks at *ψ* = 60°, characterized by L_P-PE_ ~ 180 Å. It suggests that few lamellae tilt at ±30° with the TD direction. These diffusions are correlated to diffusion observed along the TD direction in Figure 5a. The corresponding WAXS pattern (Figure 5d) shows very intense (two times more intense than in Figure 5b,f) and oriented peaks. This pattern indicates two different orientations of the PE crystalline lamellae: (i) the most important corresponds to *a*-axis along ND, *b*-axis along TD and *c*-axis along ED, and (ii) the other would be *a*-axis at *ψ* = 60° from ND, *b*-axis at *ψ* = 60° from TD and *c*-axis along ED. The *c*-axis orientation along ED corresponds to the crystalline lamellae perpendicular to ED, already observed in Figure 5a,e. Only two different orientations of the crystalline lamellae are possible: (i) the major one with the (200)_PE_ planes parallel to the PE/PA6 interfaces (i.e., parallel to the film surface), and ii) the other (in minority) with the (110)_PE_ planes parallel to the film surface.

For PA6, only γ-phase peaks at d_020_~8 Å and d_001_/d_200_/d_201_~4 Å are distinguishable, respectively associated to (020) and (001), (200) or (20$\bar{1}$) crystal planes. In ND (Figure 5a) and TD (Figure 5f), the (020) plane is oriented at *ψ* = 90° (Figure S4). This corresponds to *b*-axis chain orientation in ED for PA6, in agreement with classic on-edge lamella orientation for PA6 thin films [38,39].

### 3.2. Transport Properties

Transport properties were determined from gas (N_2_, O_2_, He and CO_2_) and water permeation measurements performed on PE, PEgMa and PA6 films (Table 2), taken as references, and on the different PE/PEgMA/PA6 multilayer films. The gas and water permeability coefficients found for PE and PEgMA for 0 and 8 LME films are similar (Table 2) and consistent with literature data [40,41,42]. In case of PA6 films, if permeability values for gases are also close between 0 LME and 8 LME and in agreement with literature [43,44,45], a surprisingly high difference for water permeability (4128 and 6653 barrer, respectively) values is obtained. In literature, water permeability of PA6 can vary between 5480 [46,47] and 11225 barrer [48]. Molecular weights measured by GPC of 0 and 8 LME films are close to those of pristine pellets, suggesting no significant degradation of the macromolecular chains of PA6 after extrusion. In addition, IR and NMR analysis were performed (data not shown), confirming no chemical modification of PA6 due to the coextrusion process. As the crystallinity was not really changed, one possible explanation for these differences in water behavior of PA6 could be related to different organization of hydrogen bonds, dependent on the presence of residual water and/or stresses exerted by the extrusion The macromolecular orientation of the amorphous phase possibly induced by the process could, impact the water sorption, as it has previously been observed for PA [47] (Table 2). Moreover, it has been shown that the barrier properties of PA6 can be dependent on the nature of the crystalline phase and its orientation. Olivier et al. [23] have shown that the γ-phase contribution to the crystalline phase of PA6, *f_γ_*, is very dependent on the films process and the gas permeability coefficients varies with the product *Xc.f_γ_* while the degree of crystallinity *Xc* of PA6 is not really changed.

To summarize permeation results, it can be stated that: (i) PE and the compatibilizer (PEgMA) exhibit similar gas and water behaviors which are not influenced by the process, (ii) PE and PA6 have complementary barrier properties (moisture resistance for PE and gas barrier properties for PA6) and (iii) the flow in the multiplying elements only alter the water permeation properties of PA6.

**Figure 6:**
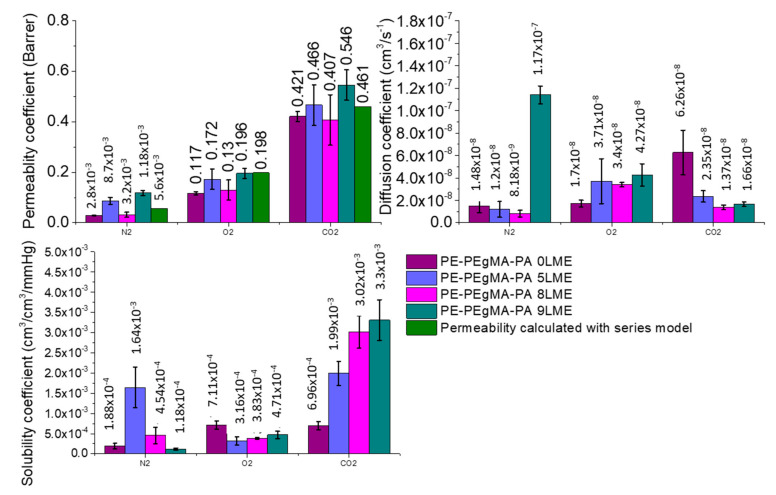
Transports properties of multilayer films compared to predicted values given by serial model.

The gas and water permeability coefficients of PE/PEgMA/PA6 multilayer films extruded with 0, 5, 8 and 9 LME were then determined (Figure 6).

To analyze the influence of the confinement effect induced in very thin layers of films on the barrier properties, experimental results were firstly compared to predicted permeabilities on the basis of the simple serial model representing the multilayer structure [17]. In the case of PE/PEgMA/PA6 films, this model gives:

(4) $$\frac{e_{film}}{P_{film}}=\frac{e_{PE}}{P_{PE}}+\frac{e_{PEgMA}}{P_{PEgMA}}+\frac{e_{PA6}}{P_{PA6}}$$

where *e*_PE_, *e*_PEgMA_ and *e*_PA6_ are the total layer thickness of each polymer and *P*_film,_ *P*_PE_, *P*_PEgMA_ and *P*_PA6_ the permeability coefficient of the multilayer film and of each polymer phase, respectively.

**Figure 7:**
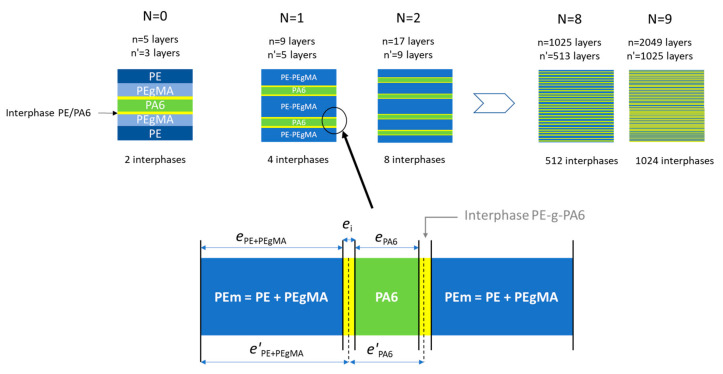
Schematic representation of the three phases (PE/interphase/PA6) model for permeation. (N = number of LME, n = the theoretical number of layers, n′ = the number of layers assuming PE and PEgMA as one phase that is PE).

As discussed previously, the polar maleic anhydride cycles of PEgMA in contact with PA6 chains react with NH_2_ amines end-groups [49,50] inducing the formation of PE-g-PA6 copolymers at the PE/PA6 interface (yellow layer in Figure 7). The resulting interphase that can be observed in Figure 2f and estimated to ~40 nm, was considered as a new resistance in the permeation model as proposed in Figure 7.

Consequently, the serial model applied to our multilayer is expressed as:

(5) $$\frac{e_{film}}{P_{film}}=\;\frac{e_{PE_{m}}}{P_{PE_{m}}}+\frac{e_{PA6}}{P_{PA6}}+\frac{e_{i}}{P_{i}}$$

With *P_PEm_* and *P_i_*, the permeability coefficients of the mixed *PE* phase (PE/PEgMA) and of the interphase *PE_m_*/*PA6*, respectively and *e_PEm_*, *e_i_*, their corresponding thickness.

In a first simple approach, it can be considered for a miscible polymer blend that the permeability coefficients *P_PEm_* and *P_i_* approximately obey to the semi-logarithmic additivity rule [51,52] of *PE/PEgMA*, and *PE_m_/PA6* respectively, as suggested by Liu et al. [47] and Zhang et al. [17]

(6) $$P_{PE_{m}}=exp^{\left[\Phi_{PE}.ln\left(P_{PE}\right)+\Phi_{PEgMa}.ln\left(P_{PEgMa}\right)\right]}$$

(7) $$P_{i}=exp^{\left[\Phi_{PEm}.ln(P_{PEm}\right)+\Phi_{PA6}.ln\left(P_{PA6}\right)]}$$

The volume fractions (*Φ*_PEm_, *Φ*_PA6_ and *Φ*_i_) are directly proportional to the layer thicknesses *e_PEm_*,/*L_film_*, *e_PA6_* /*e_film_* and *e_i_*/*e_film_*.

As the cumulated total thickness (*e_i_*) of interphase varies with the total number layers inside the films, the *PE_m_* and *PA6* layer thicknesses can be deduced according to the following equations:

(8) $$e_{PE_{m}}=e\prime_{PE_{m}}-2^{N}e_{i}$$

(9) $$e_{PA6}=e{’}_{PA6}-2^{N}e_{i}$$

With *N* the numbers of multiplier elements (LME) and *e’* the total theoretical thickness of *PE_m_* or *PA6* without interphase.

For comparison, the predicted values of permeability have been calculated without (Equation (4)) and with interphase (Equation (5)) (Table 2).

When comparing experimental data with predicted permeability coefficients (Table 2), it can be deduced that the gas barrier properties of multilayer films obtained with 8 LME shows practically the same barrier properties compared to 0 LME films, with similar BIF (Barrier improvement factor) values, (≈50% N_2_; ≈40% O_2_; and ≈10% CO_2_) if we consider the material without interphase (Equation (4)). Still, when considering the presence of an interphase (Equation (5)), the 8 LME films show higher BIF values.

**Figure 8:**
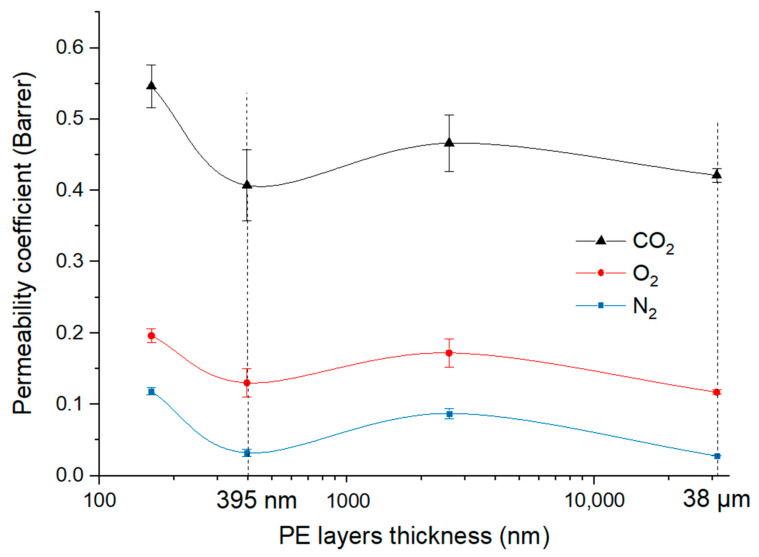
The gas permeability of PE/PEgMA/PA6 multilayer films as a function of PE (mixed PE/PEgMa) layer thickness.

If we plot the gas permeability as function of the PE layer thickness, it is interesting to see similar curves for the three gases. In order to show the possibility of thickness variations in the films we calculate the permeability coefficient for the maximum deviation of average measured thickness and report this impact on our measurements. Taking into account the measurement errors, a tendency appears for the three curves showing a minimum of permeability (Figure 8) obtained for 8 LME and for 0 LME films, in which the theoretical PE nominal layer thickness is ~300 nm (395 nm measured) and ~38 µm (32 µm measured), respectively.

Zhang et al. [17] have studied the gas and water barrier properties of confined morphology of HDPE in multilayer films. They also observed a minimum of oxygen permeability when the PE layer thickness was at ~290 nm. As the HDPE crystallinity remains constant whatever the layer thickness, these improved gas barrier properties were linked to HDPE confined crystalline morphology in the layers. According to the authors, at this thickness, spherulites disappear because they are squeezed into distorted or lamellae bundles, leading to increased tortuosity. Similarly to what we observe for 9 LME films, they show that when the HDPE layer thickness is reduced even more, permeability does not continue decreasing. From WAXS analysis, they explain this last result by the on-edge orientation of HDPE crystals which occurs when the PE layer thickness is lower than 200 nm, which is not favorable for gas barrier properties.

In our case, the situation seems rather similar for PE layers. Indeed, we have also observed that when the layers become very thin, the coextrusion process leads to on-edge orientation PE crystals while the degree of crystallinity is not really changed. For the thinnest layers (9 LME films), the loss of barrier effect (higher permeability coefficient) compared to the multilayer 8 LME films could be due not only to the on-edge orientation but also to the increase in layer breakups [10].

In the case of 8 LME films, we suppose that the improved gas barrier was limited because for such thicknesses (PE layers ~300 nm and PA6 layers ~90 nm), two antagonist effects occurs. On the one hand, spherulites have been transformed from 3-D form into 2-D form due to confinement effect, and so behave like impermeable thin discs that increase tortuosity. On the other hand, the on-edge orientation of lamellae can facilitate the migration of small diffusing species because of relatively larger voids between lamellae [17] and in the direction of the diffusion pathways.

The fact that 0 LME films exhibit similar barrier properties as 8 LME films can be explained by the difference in morphology (3D spherulites in 0 LME more favorable for gas barrier properties than on-edge orientation in 8LME films) and by a possible change in chain segment mobility. However, in addition to the morphology, the interphase can also play a role on the barrier properties and especially when its fraction increases with multiplier elements. Therefore, the volumetric fraction of interphase is much higher for 8 LME films compared to 0 LME films and can contribute to the barrier effect. Nevertheless, it is difficult to predict how and at which point these interphases can act on the barrier properties, since free volume, chain-segment mobility or crystallinity can all be modified in this region. Therefore, a first simple approach already discussed in the literature is to consider in the interphase region a polymer blend with intermediate properties.

**Figure 9:**
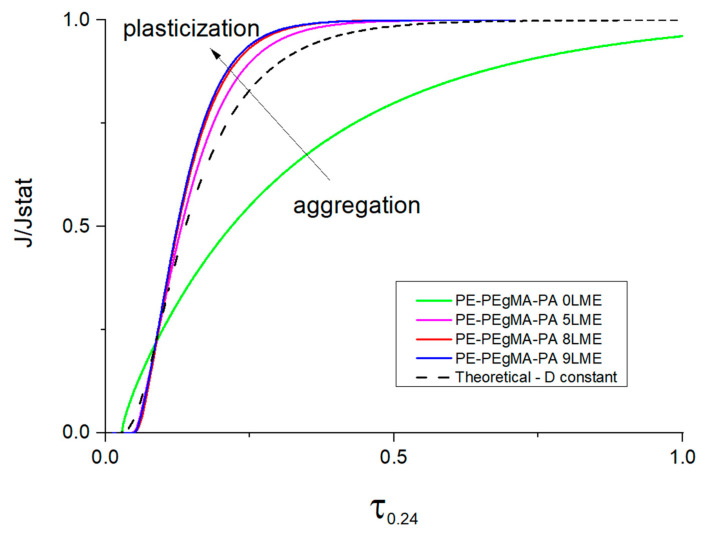
Reduced water flux through PE/PEgMA/PA6 multilayer films.

For water, the values of permeability coefficients are quite similar for all multilayer films that is to say that no enhanced barrier effect was obtained towards water molecules contrary to gas molecules. The case of water permeation is more complex than gases as water molecules can induce interactions (water/water, water/polymer) which lead to various diffusion mechanisms depending on the nature of the polymer. It is well-known that the plasticization effect of water induced in hydrophilic polymers such as PA6 allows an increase of the diffusion coefficient [44,48] due to the increase of free volume while in hydrophobic polymers such as PE, the water diffusivity is decreased with water concentration due to water aggregation (clustering phenomenon) [48]. To highlight this phenomenon in multilayers, the experimental water permeation curves have been compared to the theoretical curve assuming D constant of the Fick’s law, in the dimensionless scale of flux (J/Jst) and time (τ = Dt/L^2^) for all PE/PA6 multilayers extruded with 0, 5, 8 and 9 LME (Figure 9).

As aggregation and plasticization are antagonist phenomena on diffusion mechanism (see Figure 9), it results that the water diffusivity through the entire thickness of the PE/PA multilayer is governed by a dependence of D with C which varies differently according to the multilayer structure. Indeed, the deviation observed between the water permeation curves and the theoretical curve of Fick indicates that the water permeation through PE/PA6 multilayers is not controlled by a constant diffusion coefficient (Figure 9).

The experimental flux curve of 0 LME film is found lower than the calculated curve with D constant as permeation proceeds exhibiting a slowdown while for 5 LME, 8 LME and 9 LME films the water permeation curves are accelerated and higher than the theoretical curve. These behaviors clearly show the complexity of water diffusion mechanisms implying increase and decrease of D with C due to plasticization and aggregation (water clustering) phenomena occurring both during the permeation process. In agreement with literature, SAXS and WAXS data have shown that the reduction of the PA6 thickness leads to on-edge orientation of crystals and that can create more voids between lamellae and thus not favorable for barrier effect. Therefore, due to the high affinity of PA6 for water, this change of crystalline morphology may help water to diffuse more easily through PA6 layers. Moreover, in case of PE/PA6 multilayer everything happens as if the reduction of the layer thickness allows a better continuity of water flux passing each layer of the film. As the aggregation phenomenon is known as a slow process, it is obvious that when the thickness of the hydrophobic phase (PE) becomes thinner the water concentration profile is higher inside the layer and time necessary for cluster formation is limited so that the water flux passing through PA6 remains high at point that the water concentration inside PA layers is sufficiently high for its plasticization.

**Figure 10:**
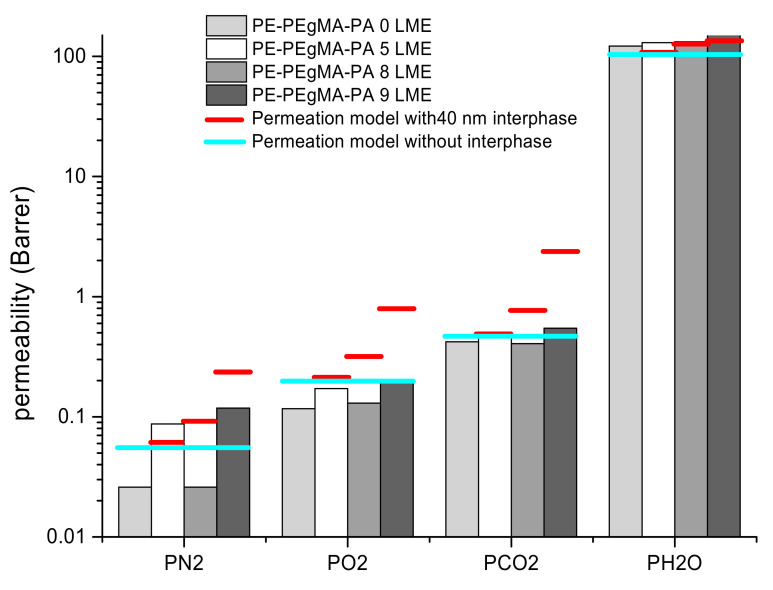
Permeability values for 25/50/25 wt % 0, 5, 8 and 9 LME PE/PEgMA/PA6 with serial model including interphase limits.

From all results, it was then interesting to compare predicted and experimental data of gas and water permeabilities. The predicted values were calculated and found higher than those calculated without interphase. The calculated permeability of the interphase (an intermediate permeability of PE and PA6, Table 2) can be subject of discussion as the interphase is a complex zone where copolymers are formed. It is clear that in multinanolayer films, the transport properties are affected by interphase which played a non-negligible role when the layer thickness varies from micro- to nano-scale. In Figure 10, despite the on-edge orientation of crystals in confined layers we note a slight improvement of the gas barrier since the permeabilities of the multilayer films obtained with 8 LME remain lower than those predicted, particularly when taking into account the 40 nm interphase.

It was found that the predicted permeability values depend highly on the consideration of the interphase so that the quality of this interphase is of prime importance and especially for multinanolayer structure in which the interphase volume fraction is not negligible. In our case, this interphase is certainly more complex than a simple blend of PEgMA and PA6 since new interactions are created leading to covalent bonds formation between both phases. It has been be deduced that the predicted permeability is higher when the interphase is considered. It results that the gas barrier improvement obtained is higher than that expected with the serial model without interphase.

As already mentioned, the stiffening of the chains would be an element of response to the increase in the barrier effect.

### 3.3. Mechanical Properties

**Figure 11:**
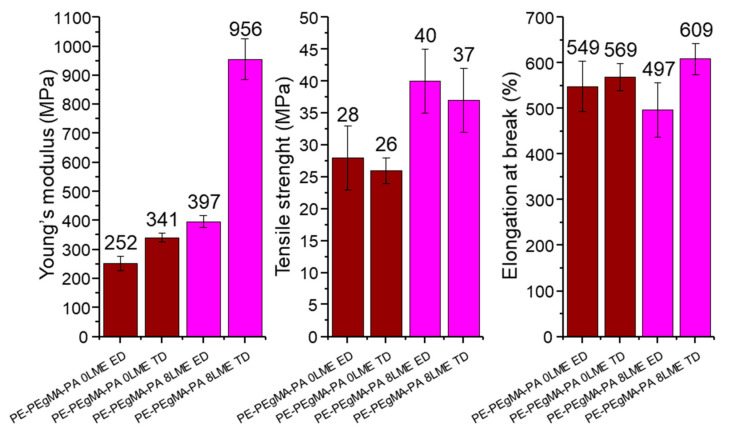
Mechanical properties in extrusion direction (ED) and transverse direction (TD) of PE/PEgMA/PA6 multilayer films extruded with 0LME (5 layers) and with 8LME (1029 layers).

The Young’s modulus, maximal strength and elongation at break have been determined for 0 and 8 LME multilayer films in ED and ND (Figure 11). The results show significant differences between the films extruded with 0 LME (5 layers) and with 8 LME (1029 layers). Without LME (0 LME), when thickness of PE and PA6 layers are in the micro-scale, the mechanical properties in ND and TD are relatively similar indicating isotropic properties and so homogenous materials. When using 8 LME, we can see an increase of the stiffness and especially in transverse direction in which the Young’s modulus is two times higher in comparison with extrusion direction. This result could be due to the effect of the reduction of layer thickness from micro-scale to nano-scale on crystal orientation and consequently the on-edge orientation of crystals in confined PE and PA6 layers. Moreover, it seems that the interphase volume fraction influences the mechanical properties as we can observe an increase of the maximal strength when the number of layers increases. If the compatibilized surface area between PE and PA6 is increased by multiplying layers in the multilayer, concerning the elongation at break no effect of the layer thickness is observed, so it can be assumed the strength properties were set by the PA6 phase while the ductility of the multilayer film is controlled by the PE phase.

From these mechanical results, as the increase in Young’s modulus with the multiplication of layers clearly shows an increase in chain stiffness, so the reduction in chain segment mobility should make the passage of molecules more difficult. Thus, the constrained amorphous phases would lead to more rigid macromolecular chains forcing the molecules to borrow more tortuous paths.

## 4. Conclusions

The use of the forced assembly coextrusion process with layer multiplying elements (LME) allowed to create multinanolayer PE/PA6 films well-structured with continuous layers down to ~50 nm. Regarding to the morphological and structural analyses, the gas barrier performances were maintained or even improved while the on-edge crystalline orientation induced in confined layers should increase the permeability. The experimental results of permeability have been compared to predicted permeabilities according to a serial model of diffusion resistances in order to see at which point the thickness decrease of confined layers can modify the barrier behavior of these multilayers. The interpretation of all permeation data has clearly shown the crucial role of the interphase PEgMA/PA6 but also the complexity of the diffusion mechanisms involved in these systems.

The increased stiffness related to confinement and multiplied interfaces can explain in part the barrier effect by the reduction of the molecular mobility, which hinders the passage of the diffusive molecules. However, as already observed in literature, there is a critical thickness threshold for the confined layers around 100 nm in our PE/PA6 multilayers, below which a loss of barrier properties appears due to defects and layer ruptures. If the multinanolayered structure of PE/PA6 allows a gas barrier improvement, in case of water no effect was observed. Interactions induced by water in polymers lead to complex diffusion mechanisms such as plasticization and clustering phenomena involving dependent-diffusion concentration.

The high quality of these PE/PA6 multinanolayer films has been shown from optical (transparency of films), barrier and mechanical properties.

In our PE/PA6 multilayer systems, the barrier improvement was limited because the on-edge orientation obtained in confined layers of both polymers, PE and PA6, is not favorable to increase tortuosity. As suggested by TM-DSC, SAXS and WAXS analyses, a thermal treatment applied with specific conditions on the multilayer films above the PA6 glass transition could further improve the barrier properties. In this way, it would be also interesting to bring rigidity in both confined polymers by incorporating nanofillers in order to favor in-plane orientation of crystals and increase tortuosity by the presence of high aspect ratio nanofillers.

## Figures and Tables

**Table 1:** Melting temperatures, melting enthalpy and degree of crystallinity determined from PE/PEgMA/PA6 films by DSC analysis.

PE/PEgMA/PA6	Tm_max_ (°C)		ΔH (J/g)		Xc (%)	
	PE	PA6	PE	PA6	PE	PA6
5 layers (0LME)	124	220	726	162	38 ± 5	30 ± 5
129 layers (5LME)	124	220	719	131	38 ± 5	24 ± 5
1025 layers (8LME)	124	219	735	207	39 ± 5	38 ± 5
2049 layers (9LME)	123	218	617	96	40 ± 5	22 ± 5

**Table 2:** Experimental and predicted values of gas and water permeability coefficients for PE, PEgMA, PA6 with 0LME and PE/PEgMA/PA6multilayer films extruded with 0, 5, 8 and 9 LME. The predicted values are calculated from the serial model without (Equation (4)) or with an interphase (Equation (5)).

			Permeability Coefficients (Barrer *)			
			N_2_	O_2_	CO_2_	H_2_O
PE 0LME (5 Layers)			1.78	5.55	23.9	91
PE 8LME (1025 Layers)			1.73	5.42	22.7	91
PEgMA 0LME (5 Layers)			1.2	3.77	16.6	81
PEgMA 8LME (1025 Layers)			1.54	4.92	20.2	83
PA6 0LME (5 Layers)			0.015	0.038	0.089	4128
PA6 8LME (1025 Layers)			0.013	0.035	0.08	6653
PE_m_/PA6 interphase (predicted)			0.12	0.41	1.42	430
PE_m_ = mixed PE/PEgMA (predicted)			1.46	4.57	19.9	89
PE/PEgMA/PA6 multilayers						
0LME (5 Layers)	Experimental		0.028 ± 0.001	0.117 ± 0.006	0.421 ± 0.020	122 ± 38
	Predicted (Equation (4))		0.056	0.199	0.462	112
	Predicted (Equation (5))		0.056	0.200	0.463	113
BIF ** (%)			50 ± 2 50 ± 2	41 ± 3 41 ± 3	9 ± 4 9 ± 4	−9 ± 34 −8 ± 34
5LME (129 Layers)	Experimental		0.087 ± 0.014	0.172 ± 0.040	0.466 ± 0.080	130 ± 46
	Predicted (Equation (4))		0.056	0.199	0.462	112
	Predicted (Equation (5))		0.059	0.209	0.487	114
BIF ** (%)			−55 ± 25 −47 ± 23	14 ± 20 18 ± 19	−1 ± 17 4 ± 16	−16 ± 41 −14 ± 40
8LME (1025 Layers)	Experimental		0.032 ± 0.010	0.130 ± 0.040	0.407 ± 0.100	132 ± 54
	Predicted (Equation (4))		0.056	0.199	0.462	112
	Predicted (Equation (5))		0.091	0.319	0.775	123
BIF ** (%)			43 ± 18 65 ± 11	35 ± 20 59 ± 13	12 ± 22 48 ± 13	−18 ± 48 −7 ± 44
9LME (2049 Layers)	Experimental		0.118 ± 0.010	0.196 ± 0.020	0.546 ± 0.060	163 ± 32
	Predicted (Equation (4))		0.056	0.199	0.462	112
	Predicted (Equation (5))		0.236	0.790	2.394	134
BIF ** (%)			−110 ± 18 50 ± 4	2 ± 10 75 ± 2	−18 ± 13 77 ± 3	−46 ± 29 −22 ± 20

* 1 Barrer = 10^−10^cm^3^(STP)·cm·cm^−2^·s^−1^·cmHg^−1^. ** Barrier improvement factor, BIF = (Pc/Pe−1)*100, Pc and Pe the calculated and experimental permeability coefficient values.

## Data Availability

Data presented in this study are available on request from the corresponding author.

## Supplementary Materials

The following are available online at https://www.mdpi.com/2077-0375/11/2/75/s1, Figure S1: PE; PEgMA; PA6 and multilayers film 8M TM-DSC thermograms left reversible heat flow right, non-reversible heat flow; Figure S2: 2D SAXS and WAXS patterns of 0 (a) and 8 (b) LME PE/PEgMA/PA6 multilayer films in normal (ND); Figure S3: 2D WAXS patterns (a) and 1D diffractograms at low (b) and high (c) scattering vector values q (or 2) of a reference PA6 film in normal (ND) at different temperatures T; Figure S4: 2D SAXS (on the left, a, c and e) and WAXS (in the right, b, d and f) 1D diffractograms integration at various azimuthal angles at room temperature of 8 LME PE/PEgMA/PA6 multilayer films in normal (ND) (a) and extrusion (ED) (b) and transverse (TD) direction; Figure S5: raw data of mechanicals tests performed on PE-PEgMA-PA6 films in extrusion and transverse direction. Table S1: main characteristics of PE, PEgMA and PA6 polymers; Table S2: thickness of multilayer films composed of 5 layers (0LME) 129 (5LME) 1024 (8LME) and 2049 (9LME) layers; Table S3: Interplanar distances, long periods and melting points of PE and PA6.
