# Supplementary material for: Structural and Barrier Properties of Compatibilized PE/PA6 Multinanolayer Films
Source: Membranes (Basel). 2021 Jan 20;11(2):75. doi: 10.3390/membranes11020075 (PMC7909415; doi:10.3390/membranes11020075)
Supplement: Supplementary file 1 [file membranes-11-00075-s001.pdf]

Supplementary Information

The density ρ, the molar mass in weight M_w and in number M_n, the apparent crystallinity ratio χ_c, and the Newtonian viscosity η^*_0 at 240°C are reported in Table SI 1. In more details, the Gas Permeation Chromatography tests for PE and PEgMA were performed at 135°C in 1, 2, 4-trichlorobenzene (TCB) [1] and for PA6 was done at 35°C in 1,1,3,3,3-hexafluoro-2-propanol (HFiP) [2]. The rheological frequency sweep tests were performed using Anton Paar MCR 502 rheometer equipped with a plate/plate geometry at γ = 1%, in the linear domain, from ω = 100 to 0.01 rad.s^{-1}, under nitrogen flow.

Table SI 1: main characteristics of PE, PEgMA and PA6 polymers

**Polymer**	**ρ [g.cm^{-3}]**	**M_w [g.mol^{-1}]**	**M_n [g.mol^{-1}]**	**η^*_0 (240°C) [Pa.s]**
**PE**	0.92	122,000	24,000	~ 20,000
**PEgMA**	0.92	85,000	21,000	~ 2,000
**PA6**	1.18	124,000	55,000	~ 5,000

Table SI 2: thickness of multilayer films composed of 5 layers (0LME) 129 (5LME) 1024 (8LME) and 2049 (9LME) layers.

**Fraction**	**LME**	**e [µm]**	**$n_{tot\ PA6}$**	**n_{PA6}**	**$e_{th\ PA6}$ [nm]**	**e_{PA6} [nm]**	**σ_{PA6} [nm]**	**n_{PE}**	**$e_{th\ PE}$ [nm]**	**e_{PE} [nm]**	**σ_{PE} [nm]**	**$\phi_{th\ PA6}$ [%vol]**	**ϕ_{PA6} [%vol]**
**25/50/25**	**0**		**1**	4	23,630	21,720	3,290	8	38,180	31,270	1,600	23.6	25.8
**25/50/25**	**5**	**100**	**32**	20	738	766	200	14	2315	2590	500	23.6	23.5
**25/50/25**	**8**		**256**	68	92	119	51	71	298	395	117	23.6	23.1
**25/50/25**	**9**		**512**	70	46	46	17	69	149	162	38	23.6	22.1

The theoretical thicknesses were obtained with the followed equation:

$$e_{thx} = \frac{e_{tot}\,\Phi_x}{n_x} \qquad \text{Eq SI 1}$$

The measured thickness is obtained by averaging the value obtained for n measured layers. For example, the PA6 thickness in 5LME films is obtained by taking the average of 20 PA6 layer thicknesses measurement.

PA6 TM-DSC thermogram during first heating shows a large exothermic variation between 30 and 140 °C, corresponding to the main physical amorphous chain relaxation (glass transition) and water evaporation (Figure SI 1), roughly estimated to ~6 % in PA6 film. The reversible flux highlights the glass transition while the non-reversible flow shows the water evaporation, at ~100 °C. The glass transition temperature T_g is estimated to ~50 °C, in agreement with literature data [3].

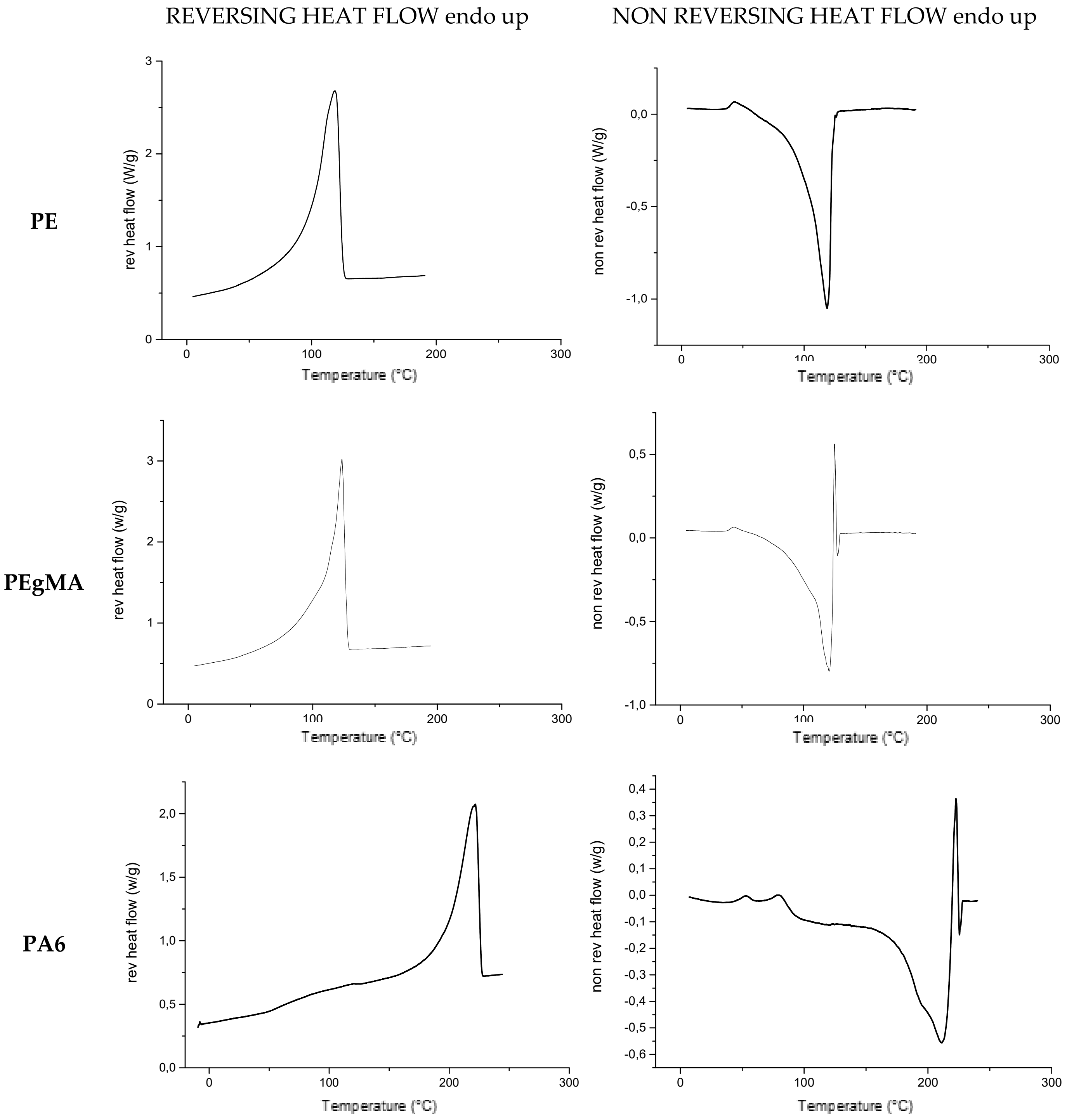

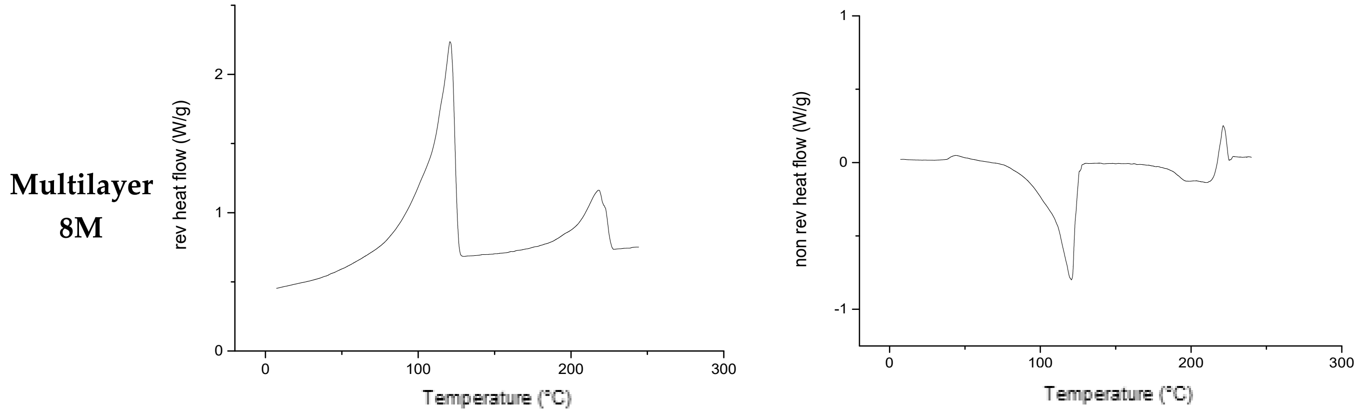

Figure SI 1: PE; PEgMA; PA6 and multilayers film 8M TM-DSC thermograms left reversible heat flow right, non-reversible heat flow

Figure SI 2 shows the SAXS and WAXS diffractograms of 0 (a) and 8 (b) irradiated at $\lambda = 0.775$ Å with X-ray beam along ND at $T = 25°C$. At this temperature, the high concentration and the strong intensity of PE patterns hide those of PA6, as observed by Dencheva *et al.* [4]. The 2D SAXS and WAXS patterns exhibit the quasi-isotropic orientation of orthorhombic peaks of PE for the 0 LME film (Figure SI 2.a), while they are highly oriented for the 8 LME film (Figure SI 2.b). Based on the intensity I azimuthal Ψ profiles obtained from SAXS patterns at $T = 25°C$, the Herman's orientation function [5] was calculated using the following equations:

$$< cos^2\Psi >= \frac{\int_0^{\pi/2} I(\Psi) cos^2\Psi sin\Psi d\Psi}{\int_0^{\pi/2} I(\Psi) sin\Psi d\Psi} \qquad \text{Eq SI 1}$$

and $$f_H = \frac{3<cos^2\Psi>-1}{2} \qquad \text{Eq SI 2}$$

where $f_H = 0$ for isotropic crystal orientation while $f_H = -0.5$ (1) for the perpendicular (parallel) orientation of the normal to the plane with the transverse direction (TD) (the reference direction correspond to $\Psi = 0°$) . Using SAXS patterns, the Hermans function $f_H = -0.38$ (transverse direction TD as reference $\Psi = 0°$) at $q = 0.03$ Å^{-1} compared to $f_H = -0.14$ for 0 LME film.

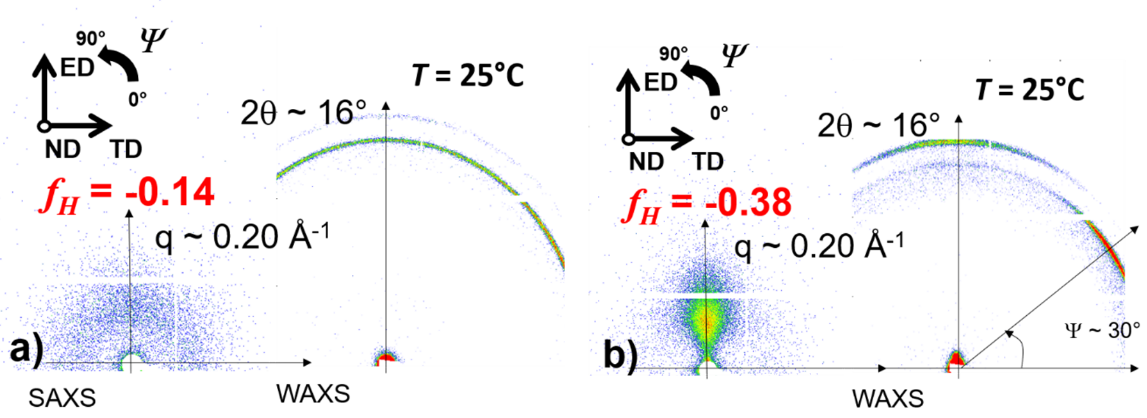

Figure SI 2: 2D SAXS and WAXS patterns of 0 (a) and 8 (b) LME PE/PEgMA/PA6 multilayer films in normal (ND)

Table SI 3 summarizes the interplanar distances, the long periods and the melting points of PE (PE and PEgMA) and PA6 polymers. Except from the peaks at ~ 8 Å and 4.4 Å the other diffraction peaks of PE and PA6 will be superimposed.

Table SI 3: Interplanar distances, long periods and melting points of PE and PA6.

	Polyethylene PE Orthorhombic[a]		Polyamide PA6 α monoclinic[b]		Polyamide PA6 γ monoclinic[c]	
			d_{020}	8.62	d_{020}	8.19
Interplanar distances (Å)			d_{200}	4.42		
	d_{110}	4.10			$d_{001}/d_{200}/d_{20\bar{1}}$	4.0-4.1
					$d_{210}/d_{011}/d_{21\bar{1}}$	3.9-4.0
	d_{200}	3.70	d_{002}	3.70		
			d_{202}	3.59		
	d_{210}	2.96				
	d_{020}	2.47				
	d_{120}	2.34	d_{402}	2.33	d_{201}	2.33
Long Period[d] (Å)	~200				~65	
Melting point Tm (°C) [**Error! Bookmark not defined.**]	112-118		220-223		214-217	

[a] *a = 7.42 Å, b = 4.95 Å and c = 2.55 Å, chains along c, from [Error! Bookmark not defined.]*
[b] *a = 9.56 Å, b = 17.24 Å, c = 8.01 Å and β = 67.5°, chains along b, from [Error! Bookmark not defined.]*
[c] *a = 9.33 Å, b = 16.88 Å, c = 4.78 Å and β = 121°, chains along b, from [Error! Bookmark not defined.]*
[d] *determined on the PE, PEgMA and PA6 reference films*

Figure SI 3 presents the evolution of WAXS patterns with X-ray along ND (a) and azimuthal integration (b) at different temperatures T (λ = 0.775 Å) for PA6 reference film using 0 LME. PA6 film is isotropic with a broad peak at small angle associated to the periodic organization of the crystalline lamellae and two diffraction lines in the wide-angle domain. A small peak attributed to the d_{020} distance at 2θ ~ 5.5° (d ~ 8 Å) and the main peak $d_{001}/d_{200}/d_{20\text{-}1}$, at 2θ ~ 11° (d ~ 4,1 Å) of the monoclinic pseudo hexagonal γ phase. During heating, this latter Bragg peak becomes sharper, and its intensity increase. This evolution suggests the presence of only γ-phase (contrary to β-phase which easily turn in α-phase [6]). We cannot calculate a crystallinity ratio with this very narrow 2θ range but the evolution of the crystalline peak ratio during heating shows a significant increase (+ 50%) between 25°C to 175°C. WAXS deconvolution was performed with Origin software using Gaussian and Pearson VII function for amorphous (which shape was fixed for all temperatures and determined in molten state) and crystalline peaks, respectively. The broad peak at low angles associated to the crystalline lamellae order increases in intensity and shifts to low angle between 70°C and 200°C. This

behavior is due to the expansion of the amorphous phase above the glass transition (Tg ~ 50 °C) and to the increase of crystallinity. Thus, the PA6 γ monoclinic crystalline phase is confirmed in the 8LME multilayer film, in agreement with DSC thermograms (Figure SI 3). The presence of γ-phase can be attributed to the rapid cooling down of films on chill rolls (T = 80°C). This fast cooling leads to the increase in crystallinity observed during heating.

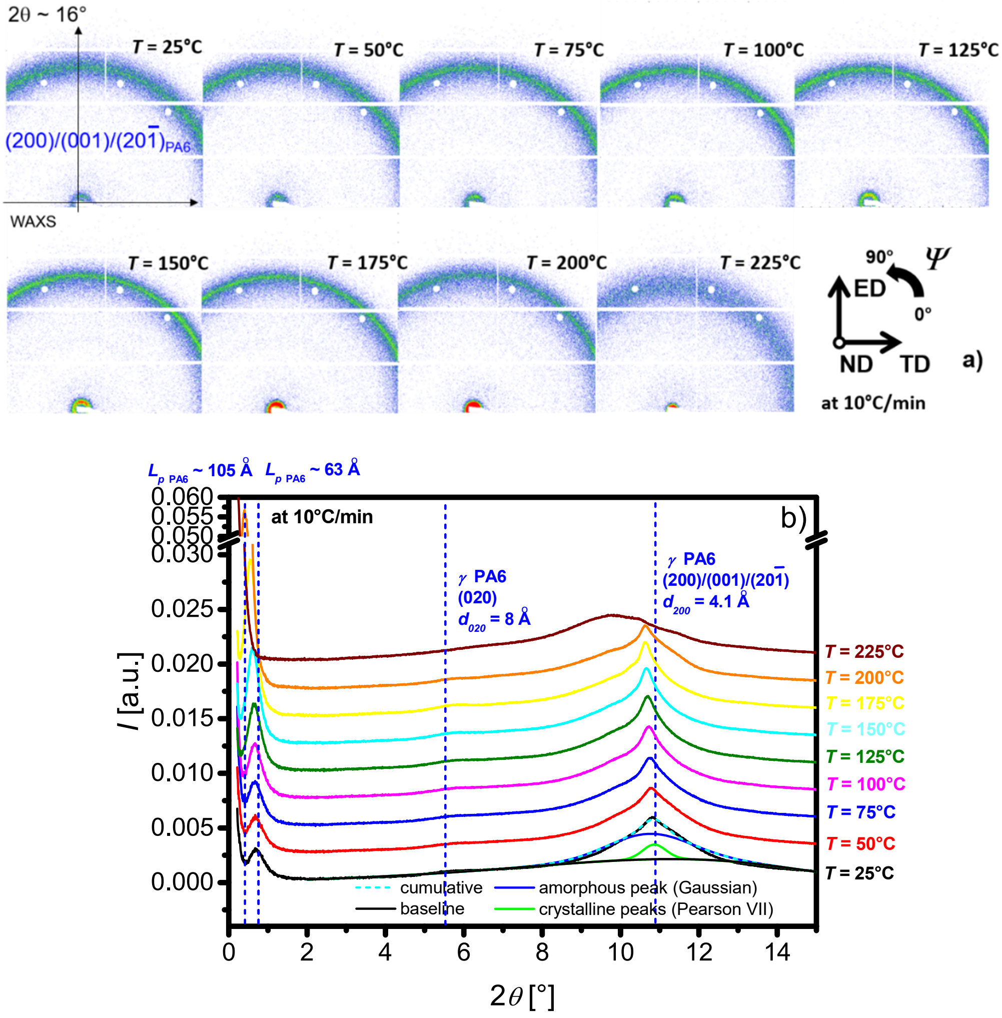

Figure SI 3: 2D WAXS patterns (a) and 1D diffractograms at low (b) and high (c) scattering vector values q (or 2θ) of a reference PA6 film in normal (ND) at different temperatures T

Figure SI 4 presents the circular 1D integration at various angle Ψ = 0, 30, 60 or 90° in ND, ED and TD. These integrations (with an angle window of 20°) were performed from the 2D images using the Foxtrot software.

These profiles help us to identify the various peaks and their respective orientation.

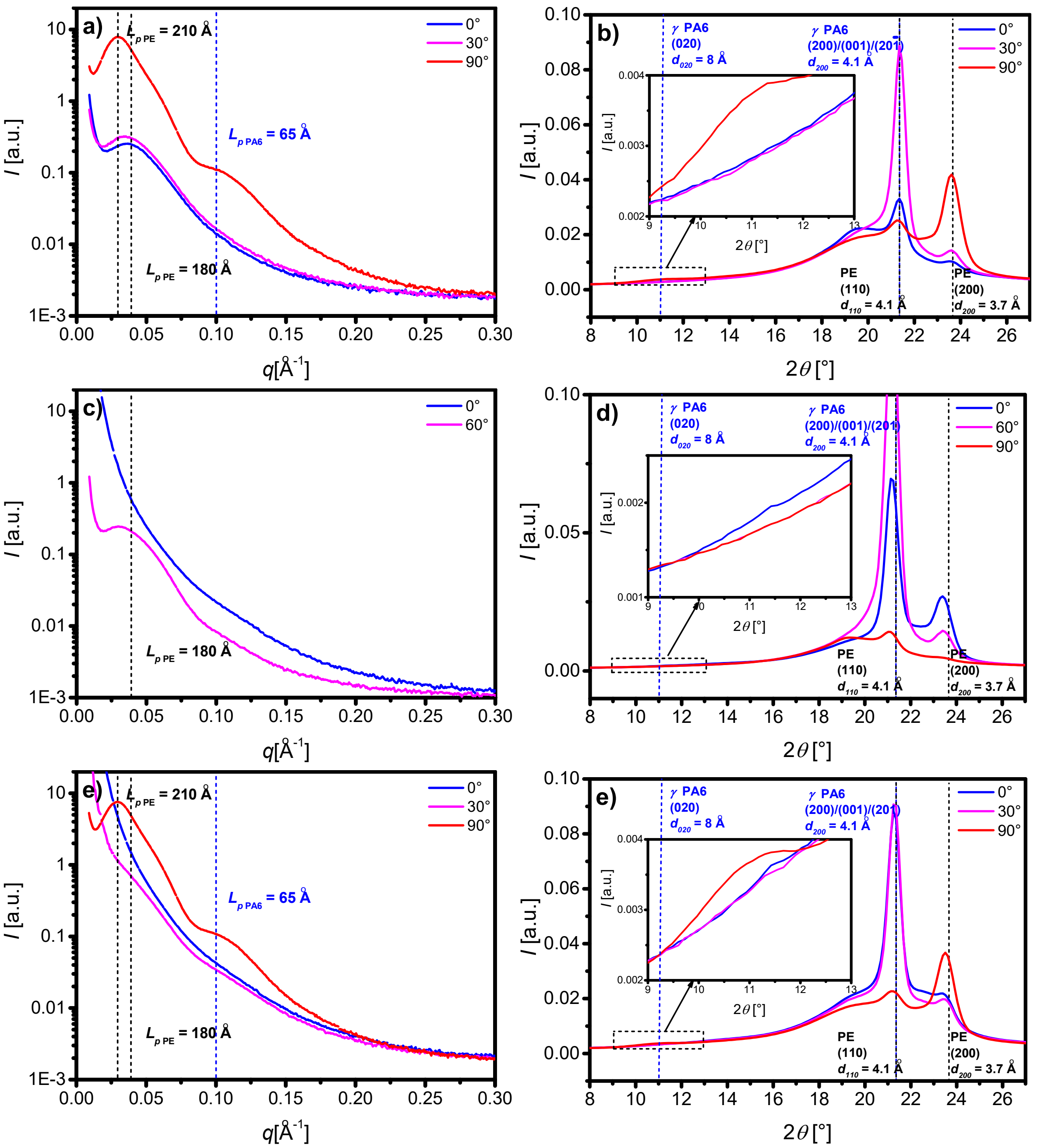

Figure SI 4:2D SAXS (on the left, a, c and e) and WAXS (in the right, b, d and f) 1D diffractograms integration at various azimuthal angles at room temperature of 8 LME PE/PEgMA/PA6 multilayer films in normal (ND) (a) and extrusion (ED) (b) and transverse (TD) direction.

Figure SI 5 represent one example of each films tested in uniaxial test in order to give an average value at least ten on each sample were tested.

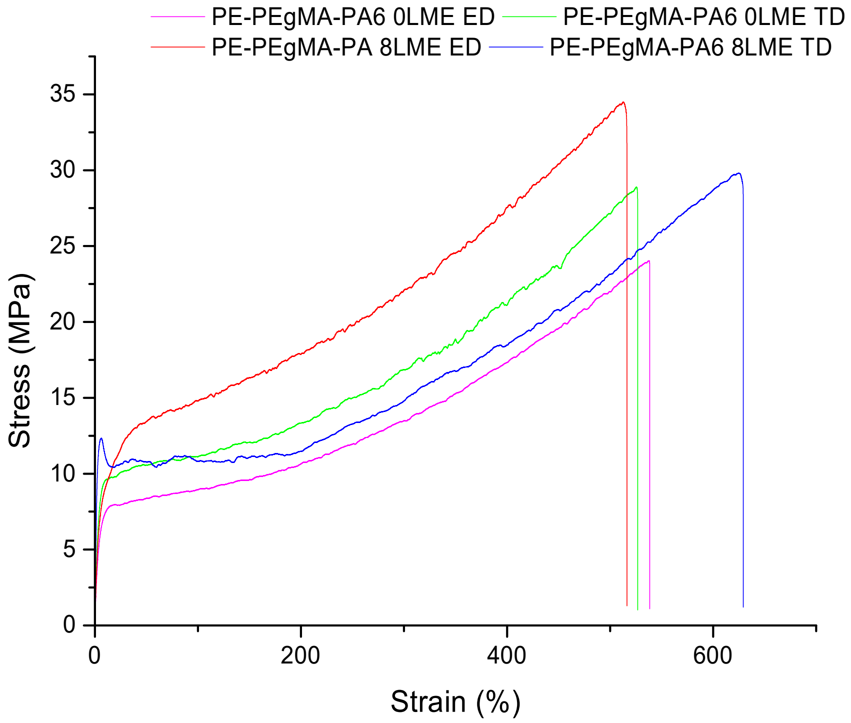

Figure SI 5: raw data of mechanicals tests performed on PE-PEgMA-PA6 films in extrusion and transverse direction.

1 Reano, A.F.; Guinault, A.; Richaud, E.; Fayolle, B. Polyethylene loss of ductility during oxidation: Effect of initial molar mass distribution. *Polym. Degrad. Stab.* **2018**, *149*, 78–84.

2 Laun, S.; Pasch, H.; Longiéras, N.; Degoulet, C. Molar mass analysis of polyamides-11 and -12 by size exclusion chromatography in HFiP. *Polymer* **2008**, *49*, 4502–4509.

3 Mark, E.J.; Polymer data handbook Oxford University Press P.181, 1999

4 Dencheva, N.; Oliveira, M.J.; Carneiro, O.S., Pouzada, A.S.; Denchev, Z. Preparation, structural development, and mechanical properties of microfibrillar composite materials based on polyethylene/polyamide 6 oriented blends. *J. Appl. Polym. Sci.* **2010**, *115*, 2918–2932.

5 Hermans, J.J.; Hermans, P.H.; Vermaas, D.; Weidinger, A. Quantitative evaluation of orientation in cellulose fibres from the X-ray fibre diagram. *Recl des Trav Chim des Pays-Bas* **2010**, *65*, 427–447.

6 Penel-Pierron, L.; Depecker, C.; Séguéla, R.; Lefebvre, J.M. Structural and mechanical behavior of nylon 6 films part I. Identification and stability of the crystalline phases. *J. Polym. Sci. Part B Polym. Phys.* **2001**, *39*, 484–495.
